# Social support and leisure-time physical activity: longitudinal evidence from the Brazilian Pró-Saúde cohort study

**DOI:** 10.1186/1479-5868-8-77

**Published:** 2011-07-26

**Authors:** Aldair J Oliveira, Claudia S Lopes, Antônio C Ponce de Leon, Mikael Rostila, Rosane H Griep, Guilherme L Werneck, Eduardo Faerstein

**Affiliations:** 1 Department of Epidemiology, Institute of Social Medicine, Rio de Janeiro State University, R Sao Francisco Xavier 524, 7th Floor, Rio de Janeiro, RJ 20550-900, Brazil; 2 Health Equity Studies Centre (CHESS), Stockholm University/Karolinska Institutet, Stockholm, Sveavägen 160, Sveaplan, Sweden; 3 Health and Environmental Education Laboratory, Oswaldo Cruz Institute, Oswaldo Cruz Foundation, Avenida Brasil, 4365, Rio de Janeiro, RJ 21045-900, Brazil

## Abstract

**Background:**

Although social support has been observed to exert a beneficial influence on leisure-time physical activity (LTPA), multidimensional approaches examining social support and prospective evidence of its importance are scarce. The purpose of this study was to investigate how four dimensions of social support affect LTPA engagement, maintenance, type, and time spent by adults during a two-year follow-up.

**Methods:**

This paper reports on a longitudinal study of 3,253 non-faculty public employees at a university in Rio de Janeiro (the Pró-Saúde study). LTPA was evaluated using a dichotomous question with a two-week reference period, and further questions concerning LTPA type (individual or group) and time spent on the activity. Social support was measured by the Medical Outcomes Study Social Support Scale (MOS-SSS). To assess the association between social support and LTPA, two different statistical models were used: binary and multinomial logistic regression models for dichotomous and polytomous outcomes, respectively. Models were adjusted separately for those who began LTPA in the middle of the follow up (engagement group) and for those who had maintained LTPA since the beginning of the follow up (maintenance group).

**Results:**

After adjusting for confounders, statistically significant associations (p < 0.05) between dimensions of social support and group LTPA were found in the engagement group. Also, the emotional/information dimension was associated with time spent on LTPA (OR = 2.01; 95% CI 1.2-3.9). In the maintenance group, material support was associated with group LTPA (OR = 1.80; 95% CI; 1.1-3.1) and the positive social interaction dimension was associated with time spent on LTPA (OR = 1.65; 95% CI; 1.1-2.7).

**Conclusions:**

All dimensions of social support influenced LTPA type or the time spent on the activity. However, our findings suggest that social support is more important in engagement than in maintenance. This finding is important, because it suggests that maintenance of LTPA must be associated with other factors beyond the individual's level of social support, such as a suitable environment and social/health policies directed towards the practice of LTPA.

## Background

Regular leisure-time physical activity (LTPA) has been linked to numerous health benefits, including decreased prevalence of coronary heart disease [1], stroke [2], high blood pressure [3], depression symptoms [4], all-cause mortality [5], and other harmful conditions [5,6]. For this reason, various demographic, psychological - and more recently, environmental and social - factors have been investigated as potential determinants of engagement in and maintenance of LTPA [7-9]. Although ongoing participation in LTPA is necessary to sustain health benefits, most studies have focused only on engagement in LTPA. A consideration of both behaviors might be relevant, because one can postulate a difference between engagement in, and maintenance of, LTPA.

Social relationships have been cited as important correlates of LTPA [10-12]. Social support and relationships can be defined as sub-concepts of social networks. In other words, social support is a social network function provided by members within a social network, and social networks generally relate to the number or frequency of contacts with family members, relatives, friends, and colleagues[13]. Social support has been defined in numerous ways, generally referring to resources supplied to individuals in need by their social network, and can be measured through the individual's perception of the degree to which interpersonal relationships can fulfill certain social support functions. Traditionally four types of social support are suggested: emotional, instrumental, appraisal, and information support [13,14]. Emotional support is most often provided by a confidant or intimate other, fosters feelings of comfort and leads an individual to believe that he/she is respected, admired and loved, and that others are available to provide love, caring and security. Instrumental or material support reflects the availability of practical services and material resources, including, for example, aid in labor, money, or kind. Information support refers to the various types of information, knowledge, and advice that are embedded in social networks [15,16]. Social network theory is based on the assumption that the network structure, by itself, is highly responsible for determining individual attitudes and behavior through access to resources and opportunities [14]. The central idea is that individuals or groups of individuals belonging to a social network provide different types of social support, and that the nature of the support given relates to the context established by the social network structure[14].

Potential mechanisms linking social relationships and long-term health consequences [17,18] have been discussed over the past few decades. Traditionally, relationships between social support and health outcomes are conceptualized in two ways: the stress-buffering model and the direct-effect model. The former model argues that social support modifies the effects of a stressful situation[19], whereas the latter suggests that social support has a beneficial impact on health, independently of the stress level[16].

Uchino [20] postulated a model in which social support may ultimately influence health through two distinct, but not necessarily independent, pathways. One involves psychological processes linked to appraisals, emotions or moods, and feelings of control. The other involves behavioral processes including health behaviors as outlined by social control and social identity theorists. According to this view, social support is health-promoting because it facilitates healthier behaviors such as engaging in physical activity, eating wisely, and abstaining from smoking. Social support can encourage individuals to initiate and maintain activities - especially LTPA - via psychological pathways including motivation and self-efficacy (indirect impact). Another mode of influence includes providing information about either the health benefits or practical aspects of the activities, and providing material resources, such as access to appropriate equipment, training facilities etc., which can increase levels of LTPA (direct impact).

In fact, social support measures have been related to increased LTPA in college students [16,21], older adults [22] and other specific populations [10,23]. Particularly in children and adolescents, the available evidence supports a causal relationship between material support and physical activity [24]. On the other hand, the literature is less clear about this relationship in the overall adult population. Although the various dimensions of social support may have varying impacts on LTPA, this is still unclear in the literature, particularly because studies are scarce, and focus mainly on the material and information dimensions [11,25]. It is also unknown whether the different dimensions of social support can influence LTPA type (individual or group). To the authors' knowledge, the present study is the first using a prospective epidemiological design to investigate the association between social support and LTPA in Latin America. The aim of this study is thus to investigate the effects of four dimensions of social support on engagement in, and maintenance of, LTPA.

## Methods

### Design and study population

The Pró-Saúde study is a prospective cohort study of socio-economic and psychosocial influences on health among non-faculty public employees at a university in Rio de Janeiro, Brazil. To date, there have been three data collection times (1999, 2001, and 2006). At time 1 (1999), all 4459 eligible workers were invited to participate, and the overall response rate was 90.4% (4030 participants); time 2 occurred in 2001. The present study was based on the 3253 subjects (1819 women and 1434 men) who participated at the first two data collection times (80.7% of 4030), with time 1 serving as the baseline for the longitudinal analyses. Employees who had retired or were on non-medical leave of absence were excluded from the analysis. Compared to Brazil's overall population, the subject group is characterized by higher levels of education and better income. Two years' follow-up will be used to evaluate engagement in, and maintenance of, LTPA. Detailed information about the cohort is available in a previous publication [26].

### Measurements

Data were gathered using self-administered questionnaires filled out in the workplace.

Questionnaires inquired about the following areas: socio-economic, demographic and psychosocial characteristics; occupational and medical history; job strain; psychological distress and stressful life events; experience with physical violence, social and racial discrimination; integration into social support webs; dietary patterns, physical activity, tobacco (active and passive) and alcohol use; history of medical diagnoses and treatments; use of medication and of unconventional therapies; practice of prevention and early diagnosis; and other behaviors and exposures with impacts on health. An average of fifty minutes was needed to fill in the questionnaire during free time provided especially for the procedure by the participant's immediate boss under an institutional agreement. Various methods were applied to ensure the quality of the information, including a large pilot study, validation of the translated scales, test-retest reliability studies, and double data entry [27,28].

Written informed consent was obtained from all participants, and the research protocols were approved by the Ethics Committee of Rio de Janeiro State University. The research was conducted in Rio de Janeiro State.

#### LTPA

LTPA was measured at times 1 and 2 as follows: respondents first answered the dichotomous question: *"In the last two weeks, have you engaged in any physical activity to improve your health, physical condition or for the purpose of fitness or leisure?"*. Respondents answering "yes" were then asked to identify the physical activity undertaken in the prior 14 days, and to quantify it in terms of duration (minutes per session) and weekly frequency. From these responses, four different outcome measures were generated: engagement in LTPA (those individuals who did not engage in LTPA at time 1, but who had become practitioners at time 2), maintenance of LTPA (those individuals who practiced LTPA at time 1 and continued practicing at time 2), type of LTPA (individual or group activity), and time spent on LTPA (per week). For example, an individual who reported two different types of activity (basketball and running) was allocated to "group activity", and the times spent performing these activities were added together to generate the time variable. Based on recommendations by the Centers for Disease Control and Prevention and the American College of Sports Medicine [29], the time spent on LTPA was dichotomized using 3 hours per week as the cut-off point. In addition, the reliability of all LTPA information was evaluated using a test-retest approach, which yielded a Kappa coefficient of 0.63 (CI = 0.54-0.73) for the filter question at time 1. Further detail is given in a previous publication[30].

#### Social support

Social support was measured by means of the Medical Outcomes Study Social Support Survey (MOS-SSS), a 19-item questionnaire that covering multiple dimensions of social support, and designed to be easily administered [15]. The items in this instrument do not specify the source of support (e.g., whether from family, friends, community or others), and they measure perceived availability of functional support. Originally designed in English, the MOS-SSS has been submitted to a process of translation and adaptation to Portuguese. This Portuguese version has shown good psychometric properties[31]. Test-retest reliability was consistently high for the subscales of the instrument (with intraclass correlation coefficients ranging from 0.78 to 0.87), and internal consistency, as assessed by Cronbach's alpha, ranged from 0.75 to 0.91. Although there are five theoretical dimensions to the MOS-SSS, previous validity investigations [15,31] have suggested that questions related to emotional and information support were grouped in the same dimension. Accordingly, the present study used four dimensions: material support, affective support, emotional/information support and positive social interaction.

#### Covariates

Socio-economic and demographic variables (age, gender, schooling, per capita household income), self-reported morbidity, tobacco and alcohol use were used as covariates in the models. Age was categorized into five groups: 20 to 29, 30 to 39, 40 to 49, and 50 or more. Household per capita monthly income was calculated as total family income divided by the number of family members living on that income, and then categorized in terms of Brazil's minimum wage. Education was measured using the Brazilian educational system and categorized into three levels: elementary (up to 6 years), secondary (up to 12 years), and higher (more than 12 years). Physical morbidity was assessed through self-reports based on a list of seventeen common diseases, and was evaluated as a dichotomous variable (at least one reported disease or none). Tobacco use was investigated as follows: "*Do you currently smoke cigarettes?" *Alcohol consumption was investigated using a dichotomous variable based on the following question: *"In the past two weeks, have you consumed any kind of alcoholic drink?" *All these variables were evaluated as possible confounders in the associations between social support and LTPA, because they have an association with social support [32] and also influence LTPA status [33].

### Statistical analysis

Scores returned for the four dimensions of social support (positive social interaction, affective support; emotional/information support and material support) were categorized into tertiles, and analyzed as explanatory variables. The three dichotomous LTPA variables - engagement (yes/no), maintenance (yes/no), and time spent on activities (up to 3 hours per week or more) - were used as outcomes. In addition, one outcome variable (type of LTPA) was used in three categories: those individuals who did not engage or maintain a LTPA (the reference group for the analysis), practitioners of individual activities, and practitioners of group activities.

We are interested in the association between dimensions of social support and engagement in, and maintenance of, LTPA over a period of two years. Binary logistic regression models were fitted for the dichotomous outcomes, and multinomial logistic regression models were fitted for the three-category outcomes. Odds Ratios (OR) and confidence intervals (95% CI) were estimated before and after adjusting for confounders. All models were conducted in order to evaluate the role of each dimension of social support on engagement in, and maintenance of, LTPA. The fully-adjusted models included the following independent variables: social support dimensions, age, gender, education, per capita monthly income, tobacco and alcohol use and morbidity. The analyses were performed using the R software, version 2.10.1.

## Results

Subjects' average age at time 1 was 40 years (standard deviation, 8.5); 40% were in the highest category of education, and 55% were women. At baseline, 45.8% of subjects reported having done at least some LTPA in the previous two weeks. Of these individuals, 81% had performed only individual LTPA, 19% performed group LTPA and 41% practiced more than three hours per week. The median time spent on LTPA was 2.6 hours per week, and percentile 25 and 75 were 1.5 and 5.0 hours per week, respectively. After two years of follow-up, the proportions of engagement in, and maintenance of, LTPA were 25.4% and 32.7%, respectively.

Analyses based solely on the dichotomous LTPA filter question showed that the dimensions of social support were not associated with whether or not individuals had pursued any LTPA in the previous two weeks in either the engagement or maintenance situation. However, the intermediate tertile of the emotional/information dimension showed a borderline association (p < .10) with maintenance of LTPA (Table 1).

**Table 1:** Frequencies of engagement in, and maintenance of, LTPA, by dimensions of social support.

Social support (tertiles)	Leisure-time physical activity					
	
		Engagement			Maintenance	
		
	n (%)	Unadjusted OR (95% CI)	Fully-adjusted OR (95% CI)	n (%)	Unadjusted OR (95% CI)	Fully-adjusted OR (95% CI)
**Material**						
Lower	464 (25)	1.00	1.00	349 (60)	1.00	1.00
Intermediate	576 (25)	1.01 (0.8-1.3)	1.06 (0.8-1.5)	487 (64)	1.21 (0.9-1.6)	1.21 (0.9-1.7)
Upper	480 (26)	1.09 (0.8-1.4)	0.96 (0.7-1.3)	438 (61)	1.04 (0.9-1.4)	0.97 (0.7-1.3)
**Affective**						
Lower	496 (24)	1.00	1.00	371 (60)	1.00	1.00
Intermediate	307 (24)	1.00 (0.7-1.3)	0.99 (0.7-1.5)	249 (59)	0.96 (0.8-1.3)	0.90 (0.6-1.3)
Upper	714 (27)	1.17 (0.9-1.5)	1.13 (0.8-1.5)	658 (64)	1.18 (0.9-1.6)	1.13 (0.8-1.6)
**Emotional/information**						
Lower	512 (22)	1.00	1.00	361 (58)	1.00	1.00
Intermediate	529 (26)	1.23 (0.9-1.5)	1.26 (0.9-1.8)	470 (65)	1.37 (1.0-1.8)	1.35 (1.0-1.9)
Upper	475 (27)	1.31 (1.0-1.7)	1.21 (0.9-1.7)	437 (62)	1.20 (0.9-1.6)	1.02 (0.8-1.5)
**Positive social interaction**						
Lower	507 (26)	1.00	1.00	347 (59)	1.00	1.00
Intermediate	454 (22)	0.83 (0.6-1.1)	0.82 (0.6-1.2)	383 (60)	1.01 (0.8-1.2)	1.13 (0.9-1.6)
Upper	556 (27)	1.07 (0.8-1.4)	0.93 (0.7-1.3)	546 (65)	1.28 (1.0-1.4)	1.09 (0.8-1.5)

Unadjusted and Fully-adjusted Odds Ratios (OR) and respective 95% confidence intervals (95%) for the logistic regression models fitted using social support dimensions as predictors of Engagement in LTPA (reference group: individuals who were inactive at time 1 and did not change their status at time 2) and Maintenance of LTPA (reference group: individuals who were active at time 1 and changed at time 2). Pró-Saúde Study, Rio de Janeiro, Brazil (2 years of follow-up).

n(%) = Number of observations and percentages of individuals who were physically active during their leisure-time according to each level of social support dimension.

Fully-adjusted models: adjusted by age, gender, education, per capita monthly income, tobacco and alcohol use, and morbidity.

The results showed that the relationships between dimensions of social support and the LTPA outcomes were in a positive direction, such that greater support predicted participation in LTPA. As shown in Table 2, in analyses restricted to the engagement group (n = 390), all dimensions of social support, except the material dimension, are related to group LTPA (fully-adjusted model). However, in the fully-adjusted model, the material dimension increases the probability of engagement in group activities by 53% (95% CI = 0.7-3.2). Individuals in the highest tertile of the positive social interaction dimension have a 79% increase in odds of engagement in group activities compared with those who did not engage in any type of LTPA during the follow-up period. In addition, according to the fully-adjusted model, the highest tertile of affective social support are more than 2.5 times more likely to engage in group LTPA, as compared to those in the lowest tertile [tertile two vs. tertile one: odds ratio (OR) 2.34, 95% confidence interval (95% CI) 1.0; 5.8/tertile three vs. tertile one: odds ratio (OR) 2.65, (95% CI 1.8; 6.0) related type of LTPA].

**Table 2:** Frequencies of LTPA type (engagement group), by dimension of social support.

Social support (tertiles)	Type of Leisure-Time Physical Activity - Engagement group (n = 390)						
	
		%	%	Unadjusted OR (95% CI)		Fully-adjusted OR (95% CI)	
						
	n	Individual	Group	Individual	Group	Individual	Group
**Material**							
Lower	112	7	12	1.00	1.00	1.00	1.00
Intermediate	141	7	9	1.07 (0.8-1.5)	0.77 (0.4-1.4)	1.10 (0.7-1.6)	0.88 (0.4-1.9)
Upper	125	7	20	1.01 (0.7-1.4)	1.51 (0.9-2.7)	0.85 (0.6-1.2)	1.53 (0.7-3.2)
**Affective**							
Lower	115	10	7	1.00	1.00	1.00	1.00
Intermediate	72	6	17	0.85 (0.6-1.2)	2.19 (**1.1-4.5**)	0.85 (0.6-1.3)	2.34 (**1.0-5.8**)
Upper	191	9	16	1.08 (0.8-1.4)	2.07 (**1.1-3.9**)	0.99 (0.7-1.4)	2.65 (**1.2-6.0**)
**Emotional/information**							
Lower	111	7	11	1.00	1.00	1.00	1.00
Intermediate	137	9	14	1.23 (0.9-1.7)	1.37 (0.8-2.5)	1.20 (0.8-1.7)	1.77 (0.8-3.8)
Upper	129	10	15	1.31 (1.0-1.8)	1.50 (0.8-2.7)	1.05 (0.7-1.5)	2.33 (**1.1-5.0**)
**Positive social interaction**							
Lower	128	10	9	1.00	1.00	1.00	1.00
Intermediate	101	5	14	0.75 (0.5-1.0)	1.42 (0.8-2.7)	0.71 (0.5-1.0)	1.82 (0.8-4.0)
Upper	150	9	16	1.00 (0.8-1.4)	1.60 (0.9-2.9)	0.82 (0.6-1.1)	1.79 (**1.1-3.9**)

Unadjusted and adjusted Odds Ratios(OR) and respective 95% confidence intervals(95%) provided by multinomial regression models fitted using social support dimensions as predictors of type of Leisure-time physical activity (reference group: individuals who were inactive at time 1 and did not change the status at time 2). Pró-Saúde Study, Rio de Janeiro, Brazil (2 years of follow-up).

Fully-adjusted model: Adjusted by age, gender, education, per capita monthly income, tobacco and alcohol use and morbidity.

All statistically significant associations are in bold.

Analysis restricted to the maintenance group (n = 798) showed that individuals with higher levels of material and positive social interaction support had increased odds of performing a group activity as compared with those who ceased to practice a LTPA (Table 3). For instance, after adjustment for confounders, individuals in the highest tertile of the affective dimension and in the intermediate tertile of positive social interaction were, respectively, 50% and 80% more likely to perform group activities.

**Table 3:** Frequencies of LTPA type (maintenance group), by dimension of social support.

Social support (tertiles)	Type of Leisure-Time Physical Activity - Maintenance group (n = 798)						
	
		%	%	Unadjusted OR (95% CI)		Fully-adjusted OR (95% CI)	
				
	n	Individual	Group	Individual	Group	Individual	Group
**Material**							
Lower	205	9	19	1.00	1.00	1.00	1.00
Intermediate	313	10	24	1.18 (0.9-1.6)	1.39 (0.9-2.0)	1.07 (0.7-1.6)	1.80 (**1.1-3.1**)
Upper	266	8	23	0.99 (0.7-1.4)	1.27 (0.9-1.9)	0.80 (0.6-1.2)	1.50 (0.9-2.6)
**Affective**							
Lower	218	9	22	1.00	1.00	1.00	1.00
Intermediate	146	8	25	0.94 (0.7-1.3)	1.07 (0.7-1.7)	0.84 (0.6-1.3)	1.03 (0.6-1.8)
Upper	420	10	22	1.21 (0.9-1.6)	1.21 (0.9-1.7)	1.04 (0.7-1.5)	1.48 (0.9-2.4)
**Emotional/information**							
Lower	205	10	24	1.00	1.00	1.00	1.00
Intermediate	306	14	26	1.42 (**1.0-1.9**)	1.33 (0.9-1.9)	1.32 (0.9-1.9)	1.52 (0.9-2.5)
Upper	271	12	22	1.34 (**1.0-1.8**)	0.99 (0.7-1.5)	1.06 (0.7-1.5)	0.99 (0.6-1.6)
**Positive social interaction**							
Lower	202	7	20	1.00	1.00	1.00	1.00
Intermediate	228	6	24	0.93 (0.7-1.3)	1.30 (0.9-2.0)	1.03 (0.7-1.5)	1.51 (0.9-2.6)
Upper	354	11	26	1.22 (0.9-1.6)	1.51 (**1.0-2.2**)	0.97 (0.7-1.4)	1.56 (**1.0-2.6**)

Unadjusted and adjusted Odds Ratios(OR) and respective 95% confidence intervals(95%) provided by multinomial regression models fitted using social support dimensions as predictors of type of Leisure-time physical activity (reference group: individuals who were inactive at time 1 and did not change the status at time 2). Pró-Saúde Study, Rio de Janeiro, Brazil (2 years of follow-up).

Fully-adjusted model: Adjusted by age, gender, education, per capita monthly income, tobacco and alcohol use and morbidity.

All statistically significant associations are in bold.

Table 4 shows the results for the association between social support and time spent on LTPA. For the engagement group, the highest level of the material dimension and the intermediate level of the emotional/information dimension were associated with time spent on LTPA. Moreover, there was a borderline association (p < .10) with the intermediate level of the positive social interaction dimension (OR = 1.91; CI95%; 1.0-2.6). In the maintenance group, participants with high and medium levels of positive social interaction support were, respectively, 49% and 65% more likely to perform three hours or more of LTPA per week. Similar results were obtained in the middle tertile of the affective dimension (Table 4).

**Table 4:** Frequencies of more than three hours spent on LTPA per week, by dimension of social support.

Social support (tertiles)	Time on Leisure-time Physical Activity					
	
	Engagement group			Maintenance group		
		
	n (%)	Unadjusted OR (95% CI)	Fully-adjusted OR (95% CI)	n (%)	Unadjusted OR (95% CI)	Fully-adjusted OR (95% CI)
**Material**						
Lower	87 (34)	1.00	1.00	167 (55)	1.00	1.00
Intermediate	120 (43)	1.45 (0.8-2.5)	1.27 (0.7-2.0)	282 (54)	0.93 (0.7-1.3)	0.80 (0.5-1.2)
Upper	105 (49)	1.75 (**1.1-2.5**)	2.06 (**1.0-4.2**)	227 (57)	1.09 (0.7-1.6)	0.94 (0.5-1.5)
**Affective**						
Lower	93 (39)	1.00	1.00	182 (52)	1.00	1.00
Intermediate	58 (40)	1.04 (0.5-2.0)	0.80 (0.3-1.7)	130 (59)	1.36 (0.9-2.1)	1.67 (1.0-2.9)
Upper	161 (47)	1.38 (0.8-2.3)	1.24 (0.7-2.3)	365 (55)	1.14 (0.9-1.6)	1.27 (0.8-1.9)
**Emotional/information**						
Lower	90 (28)	1.00	1.00	172 (47)	1.00	1.00
Intermediate	119 (54)	2.50 (**1.6-4.0**)	2.01 (**1.2-3.9**)	269 (59)	1.62 (**1.1-2.3**)	1.45 (0.9-2.3)
Upper	102 (44)	2.00 (**1.1-3.1**)	1.62 (0.8-3.8)	235 (56)	1.43 (0.9-2.2)	1.34 (0.8-2.2)
**Positive social interaction**						
Lower	101 (34)	1.00	1.00	169 (50)	1.00	1.00
Intermediate	91 (56)	2.10 (**1.4-3.9**)	1.91 (**1.0-2.6**)	200 (58)	1.42 (**1.0-2.1**)	1.65 (**1.1-2.7**)
Upper	121 (41)	1.38 (0.8-2.4)	1.14 (0.6-2.2)	308 (56)	1.26 (0.9-1.8)	1.49 (1.0-2.3)

Unadjusted and adjusted Odds Ratios(OR) and respective 95% confidence intervals(95%) for the logistic regression models fitted using social support dimension as the predictor of time spent on Leisure-time physical activity (reference group: individuals who spent less than 3 hours per week). Pró-Saúde Study, Rio de Janeiro, Brazil (2 years of follow-up).

Fully-adjusted model: Adjusted by age, gender, education, per capita monthly income, tobacco and alcohol use and morbidity.

All statistically significant associations are in bold.

## Discussion

LTPA is a behavior that involves different types of activities (e.g., group, individual, recreational and competitive activities), which occur in different social contexts for varied lengths of time and with varied levels of physiological demands. Because of this scenario, it was decided to investigate various features of physical activity in order to understand the characteristics of the relationship between social support and LTPA better. This study examined the association of social support dimensions (i.e., material, emotional/information, affective and positive social interaction) with four LTPA outcomes (engagement, maintenance, LTPA type, and time spent on LTPA). Our results suggest that the influence of social support on LTPA depends on the social support dimension, LTPA outcomes and the group evaluated (those recently engaged or those who maintain LTPA). It is thus plausible that there are different pathways linking social support and LTPA. In our view, the material and emotional/information dimensions might be directly linked with LTPA because they relate the availability of physical activity resources and exposure to health information, respectively. On the other hand, the positive social interaction dimension might be linked to LTPA by providing motivation and self-efficacy. The role of self-efficacy as a mediator of the relationship between social support and health-related behavior has been demonstrated previously in the physical activity literature [12,34]. Moreover, several theories attempt to explain how protective behaviors are initiated or maintained. The main idea of these theories is that motivation toward protection results from a perceived threat and the desire to avoid the potential negative outcome. In other words, the motivation is related to the health and aesthetic benefits that a physical activity could provide. Thus, the positive social interaction dimension can be linked to this pathway, because it involves informal social control through norms and attitudes. It could then be related to higher or lower levels of physical activity, depending on the context established by the social network providing the social support[16]. Our results show that positive social interaction in the form of material and emotional/information supports was related to higher levels of LTPA, suggesting that members of the study population were surrounded by social networks that tend to support the practice of physical activity. On the other hand, we did not find an association between dimensions of social support and LTPA based on the filter question (whether any physical activity had been performed in the previous two weeks), a negative finding that could have resulted from the generic phrasing of the LTPA question. This finding emphasizes the importance of using more specific LTPA variables. Also, there is weak evidence of the affective dimension's influencing LTPA; only in the relationship between this dimension and LTPA type did we find a significant association. These findings may reflect the characteristics of the dimension, in that affective support may exert a more indirect influence on LTPA than the other dimensions.

In the engagement group results, all dimensions of social support are related to engagement in group activities, but not in individual activities. These results are interesting because engagement in group activities is often more difficult for the following reasons: first, accessing specific materials and locations for group activities, which could be related to material and emotional/information dimensions of social support, are the first practical steps to beginning a group activity; and, second, knowing or learning certain basic rules and techniques for the specific physical activity often requires instrumental support. However, some group leisure-time physical activities are so traditional that they are intrinsically familiar (e.g., soccer in Brazil, basketball in the United States). Finally, arranging the time for all participants to perform the activity could be a barrier. Thus, it is plausible that individuals with higher levels of social support are more likely to surpass all these barriers and join in a group activity than are others with low levels of social support. The results for time spent on LTPA are less striking than for LTPA type, although individuals with high levels of the emotional/information and positive social interaction dimensions of social support are more likely to perform more than four hours per week, as compared with the others who performed only a maximum of 2 hours per week. These findings indicate two different modes of social support: first, the influence of the emotional/information dimension on the time spent on LTPA is related to the exposure to health information that could improve knowledge of the benefits of physical activity [35]. Second, the social positive interaction dimension significantly increases the possibility that an individual will be in contact with individuals with whom to engage in leisure activities, including physical activities.

In the maintenance group, only the material dimension influenced LTPA type, and the emotional/information and social positive interaction dimensions were related to time spent on LTPA. These findings suggest that, among individuals still involved in physical activity after two years of follow-up (between 1999 and 2001), only practical aspects, such as access to appropriate materials or locations, were important to their continuing or engaging in group activities. In other words, interactions with individuals represented by the positive social interaction dimension could positively influence motivation to perform, and the sense of confidence in performing, a physical activity, which would, consequently, increase the amount of time spent on LTPA. As self-efficacy theory suggests, the information and feedback that an individual gains from performing an activity and the belief in their enhanced ability to perform the activity could be related to maintenance of the activity and the time spent performing it [36]. In addition, the maintenance group could be exposed to basic information about physical activity (e.g., time and intensity) and might perform the activities based on this information. It could be that middle and high levels of the emotional/information dimension are related to being involved in LTPA for more than three hours per week, a level that is closer to current health recommendations.

Overall, the results did not show any simple dose-response effect relating levels of social support dimensions and aspects of LTPA. Furthermore, an intermediate level of positive social interaction seems to be more important than the highest level in relation to time spent on LTPA. These findings suggest that the intermediate level of social support may be sufficient to influence LTPA and that the highest level of social support may not yield any additional impact on LTPA. It may also be that, to some extent, the highest level of support reflects the downsides of social relationships [13]. It is plausible, for instance, that highly supportive relationships sometimes provide information that discourages rather than promoting LTPA.

Despite the fact that comparisons between engagement in, and maintenance of, LTPA were not the focus of this study, it is notable that the influence of social support differs between the engagement and maintenance situations, suggesting that social support has different impacts on these groups. Our findings suggest that social support is more important to engagement in, than to maintenance of, physical activity. Nevertheless, a previous study [37] suggests that social support is equally important in both situations.

Although we did not find studies using time and type of LTFA as the main outcomes to investigate the potential influence of social support, our results are in line with previous work which observed associations between social support and LTPA, either in general population-based studies [11,38] or in specific subgroups [10,22]. For example, one study [38] found that instrumental church-based social support helped initiation of physical activity in a rural population.

Some limitations of our study should be noted. The use of self-reporting to measure LTPA and the use of a social support instrument that did not focus on LTPA may have limited the scope for comparison with other studies' findings. On the other hand, with these measurement strategies, we generated helpful LTPA outcome variables and investigated the role of all social support dimensions on LTPA. Second, time spent on LTPA, as reported in the questionnaire, may have been overestimated. However, the strategy of individuals filling in the information about time spent on LTPA separated by activity and session probably minimized this problem. Third, this is a specific occupational cohort of public employees in Rio de Janeiro, probably with higher levels of LTPA, and it is uncertain how far the findings of this study can be generalized to the overall population of Brazil or to other occupational groups and countries. Fourth, because the study design was based on access to LTPA data at only two points in time, it was not possible to evaluate for possible changes in LTPA that may have occurred during the follow-up period. Fifth, some models returned large confidence intervals of the effect measure evaluated in the study, probably due to missing values. To evaluate the impact of this problem, we performed models based on multiple data imputations and a sensitivity analysis which found similar results. Finally, another possible criticism of the study is that engagement in/maintenance of LTPA may result from health campaigns promoted by the university. However, the fact that none took place during the period covered by the study makes our results even more robust.

## Conclusion

To the authors' knowledge, the present study is the first to use a longitudinal approach to demonstrate that social support influences the type of, and time spent on, LTPA in a working population. In general, different dimensions of social support play different roles, and these roles seem to be more important for engagement in, than maintenance of, LTPA. This finding has social/health policy implications, because continuation of physical activities relates significantly to practical aspects of these activities, including environmental facilities and public policies focused on practicing LTPA. Another interesting finding is that information support has direct influence on the time spent on LTPA and, consequently, may play an important role in recommendations for the practice of LTPA. The study results showing an association between social support and LTPA among university employees underline the need for university management to show greater commitment to encouraging this practice. Incentives can be offered through more and better material structure, but also by allocating time and resources for social interaction and social relationships among university employees.

Finally, we are aware that our results do not reflect all the complexity of the mechanisms involved in the association between social support and physical activity. Accordingly, further studies should be conducted in order to understand such mechanisms.

## Abbreviations

LTPA: Leisure-Time Physical Activity; MOS-SSS: Medical Outcomes Study Social Support Survey.

## Competing interests

The authors declare that they have no competing interests.

## Authors' contributions

AJO and CSL conceived the study and participated in its design. They were also involved in analyzing data, interpreting results, writing the manuscript and constructing the final version. AMPL and MR contributed to the writing, participated in data analysis and interpretation of results. RHG was involved in the study design and operationalizing the measure of social support. GLW and EF were involved in the subsequent critical reviews designed to improve the coherence of the text. All authors contributed to preparing the manuscript and approved the final version. EF, CSL and GLW coordinated the main cohort study.
